# Analyzing and Synthesizing Phylogenies Using Tree Alignment Graphs

**DOI:** 10.1371/journal.pcbi.1003223

**Published:** 2013-09-26

**Authors:** Stephen A. Smith, Joseph W. Brown, Cody E. Hinchliff

**Affiliations:** Department of Ecology and Evolutionary Biology, University of Michigan, Ann Arbor, Michigan, United States of America; University of California San Diego, United States of America

## Abstract

Phylogenetic trees are used to analyze and visualize evolution. However, trees can be imperfect datatypes when summarizing multiple trees. This is especially problematic when accommodating for biological phenomena such as horizontal gene transfer, incomplete lineage sorting, and hybridization, as well as topological conflict between datasets. Additionally, researchers may want to combine information from sets of trees that have partially overlapping taxon sets. To address the problem of analyzing sets of trees with conflicting relationships and partially overlapping taxon sets, we introduce methods for aligning, synthesizing and analyzing rooted phylogenetic trees within a graph, called a tree alignment graph (TAG). The TAG can be queried and analyzed to explore uncertainty and conflict. It can also be synthesized to construct trees, presenting an alternative to supertrees approaches. We demonstrate these methods with two empirical datasets. In order to explore uncertainty, we constructed a TAG of the bootstrap trees from the Angiosperm Tree of Life project. Analysis of the resulting graph demonstrates that areas of the dataset that are unresolved in majority-rule consensus tree analyses can be understood in more detail within the context of a graph structure, using measures incorporating node degree and adjacency support. As an exercise in synthesis (i.e., summarization of a TAG constructed from the alignment trees), we also construct a TAG consisting of the taxonomy and source trees from a recent comprehensive bird study. We synthesized this graph into a tree that can be reconstructed in a repeatable fashion and where the underlying source information can be updated. The methods presented here are tractable for large scale analyses and serve as a basis for an alternative to consensus tree and supertree methods. Furthermore, the exploration of these graphs can expose structures and patterns within the dataset that are otherwise difficult to observe.

## Author Summary

Phylogenetic trees are the most common datatype by which we examine evolutionary patterns. However, biological and practical considerations require the exploration of other models. Here, we address a problem concerning the representation of conflicting and partially overlapping datasets in phylogenetics. We examine the problem of aligning many source trees from independent phylogenetic analyses into a structure that can be analyzed and synthesized but retain all of the original structure and source information. We present methods to map trees into a common graph structure using a graph database. This allows the information in the trees to be stored and synthesized in several ways. Specifically, we demonstrate how these graphs can be used to construct enormous trees as an alternative to labor-intensive grafting exercise and other methods that make the synthetic tree difficult to update. We also show how examination of the relationships in the graph allows patterns to emerge concerning support and information that are difficult to discern with existing methods. Because these methods scale well into the millions of nodes, these techniques should lead to the construction and maintenance of even larger phylogenies and new techniques for analyzing graphs that maintain the structure of the underlying trees.

**This is a** ***PLOS Computational Biology*** **Methods article.**

## Introduction

Evolutionary biologists use phylogenetic trees to conceptualize, visualize, and analyze the relationships among biological lineages. However, when examining trees from individual datasets (e.g., posterior distribution of trees, bootstrapped trees, individual gene trees) or multiple partially overlapping datasets, topological conflict is inevitably present. Conflict resulting from incomplete lineage sorting, horizontal gene transfer, and hybridization, as well as uncertainty due to lack of phylogenetic signal, provide reasons to consider alternatives to strictly acyclic data structures for analysis and/or visualization [1]–[3]. While trees perform well for many analytical purposes, practical and biological reasons exist to explore other potential models for encoding information about evolutionary relationships [4]. Here, we examine methods for combining trees into a graph datatype while retaining all of the original information from the source phylogenies.

The need to visualize and analyze variability among phylogenetic trees has fostered the development of many methods, including consensus trees, cloudograms, concordance analysis, bipartition support, splits graphs, and supertree algorithms [4]–[10]. Huson and Scornavacca [11] review a number of phylogenetic network methods that make use of graphs of higher complexity than strictly-bifurcating trees. Many of these attempt to infer a network structure from a sequence alignment instead of aligning source trees into a common structure. Other methods, used to identify hybridization and recombination events, recognize conflict in source trees. However, like network methods, they explicitly assume specific biological events to be the source of the conflict. Although many of these methods continue to be useful in exploring certain events, they do not fully retain the structure of the original source trees in the output statistics or summary networks.

Phylogenies can also be combined to construct a synthetic tree from source trees with partially overlapping taxon sets. Supertree methods are commonly used for this purpose [5], [12], [13]. These methods often produce a tree or trees (the supertree) intended to represent the relationships supported by the input trees. Although this has been demonstrated to be useful in many studies [14]–[16], one drawback of supertree methods is that the identifiability of the source trees themselves is lost in the supertree-building process. Furthermore, supertree methods can reconstruct relationships that are not found in any of the input trees [17] making it difficult to interpret the source for such relationships. In addition to these criticisms, supertree methods are not explicitly targeted toward the exploration of variability among the input trees. One recent supertree method [18] makes explicit use of a graph to construct a supertree from source trees that sample nodes from different taxonomic levels. The graph structure in this method functions as an temporary intermediate step. In the Berry et al. [18] graphs, nodes and edges from source trees are mapped in a way that facilitates the extraction of the supertree from the graph, but the semantic identity of nodes and edges is different in the graphs than it is in the source trees (for instance, sibling nodes in source trees are directly connected by edges in the graph). In addition to addressing supertree analyses and bootstrap or posterior probability summaries, the benefit of more generalized data structures for combining trees can simplify practical exercises such as tree grafting [19] and comparisons among trees with fully or partially overlapping tip sets.

Existing solutions to both the tree synthesis and exploration of conflict problems involve analyses that result in a tree or set of trees that contain nodes and edges that are difficult to trace back to the source. Whether because of lack of available database technology or somewhat different goals, the problem of mapping a set of trees, while retaining all of the original information, into a common structure is not often addressed.

We present a set of methods intended to facilitate generalized analyses involving potentially conflicting phylogenetic trees with fully or partially overlapping sets of taxa. These methods address the problem of identifying common nodes and edges across sets of phylogenetic trees and constructing a data structure that efficiently contains this information while retaining original source information. We achieve this with algorithms that align trees into a graph structure called a tree alignment graph (TAG) and stores this information so it can be queried. These methods, which align and identify equivalent nodes across trees or graphs, fall within a class of methods known as graph alignment, and are analogous to the alignment methods of other domains [20]–[22]. The goals achieved by aligning trees into a TAG are distinct from the amalgamation of sets of trees into a single tree (e.g. supertree methods), though, in addition to many other analyses, TAGs can be used to facilitate supertree and grafting exercises. As mentioned above, Berry et al. [18] have also examined placing trees into graphs for the specific purpose of constructing supertrees. The methods presented here are intended to be more generally applicable and provide additional means for storing and querying. Other uses of TAGs include mapping uncertainty across trees, synthesis and extraction of a diverse set of summary trees, and more extensive queries that have previously been difficult to address.

Mapping trees into a TAG exploits the fact that rooted phylogenetic trees are in fact a specific type of graph: they are directed, acyclic, and require that each node has, at most, one parent. By relaxing these requirements, we can combine multiple trees into a common graph, while minimizing changes to the semantic interpretations of nodes and edges in the trees. Because they contain nodes and edges directly analogous to those from their source trees, TAGs have the desirable quality of retaining the full identifiability of the original source trees they contain. Additionally, because they are not restricted to the bifurcating model of evolution, TAGs may represent conflict among source trees as reticulations in the graph. Despite having higher complexity than trees, the graphs we present are amenable to fast traversals and straightforward interpretations regarding the evolutionary relationships they imply. In addition to the extraction of synthetic trees by customizable queries, TAGs also support the extraction of the original source trees themselves for the purposes of further analysis or updating.

Here we provide a description of the TAG datatype and some associated analyses. We also demonstrate the alignment of disparate phylogenetic trees with partially overlapping sets of terminal nodes into a graph, the exploration of conflicting and complementary hypotheses of ancestry defined by the input trees, and the extraction of synthesized trees summarizing compatible relationships from multiple input source trees.

## Methods

**Figure 1:**
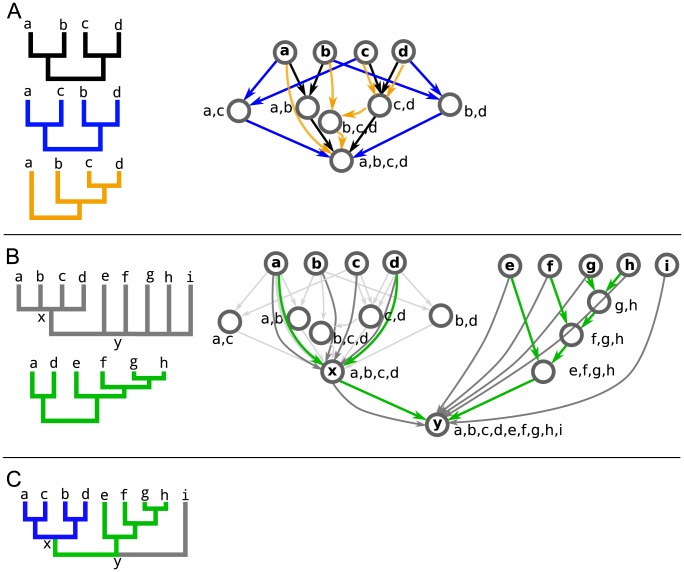
Mapping schematic. A basic schematic of the results of mapping and synthesis. A) Three source trees with completely overlapping taxon sets (left) mapped into a graph (right). The colored edges in the graph correspond to the source trees on the left, with graph nodes represented as gray circles. Internal graph nodes represent least inclusive common ancestors (LICAs), and are labelled with their descendant terminal taxa. B) A grey taxonomy hierarchy and additional green source tree added to the black, blue, and orange trees from A. The relationships presented in the black, blue, and orange trees are in light grey to cut down on clutter. C) A synthetic tree resulting from preferring source blue, green, and taxonomic source trees.

The goals of aligning trees into a common graph are to 1) efficiently store potentially conflicting hypotheses about evolutionary relationships and 2) provide a framework by which information encoded in the source trees can be synthesized to test and develop evolutionary hypotheses. There are potentially many ways in which trees can be aligned into a graph. Here we describe ways to align trees into a TAG for partially and fully overlapping taxa. We will use Figure 1 as a motivating example. First, showing the mapping of fully overlapping taxa (Figure 1A), then partially overlapping taxa (Figure 1B), and finally synthesis of the graph into a tree (Figure 1C).

### Definitions

The procedures discussed below require as input a set of rooted, acyclic, 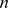-furcating source trees 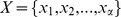, whose terminal nodes are labeled according to a common convention (see below). A source tree, 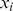, may typically be a phylogenetic tree but can be any hierarchy implying ancestry (e.g. a taxonomic classification). The complete set of source tree nodes from 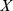 is defined as 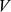, with each tree 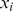 containing a set of nodes 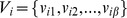. A branch extending from node 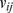 to parent node 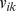 in source tree 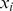 is defined as 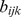. The set of all branches in the set of all sources trees 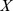 is defined as 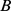.

The graph 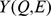 defines a common data structure into which the complete set of trees 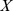 are aligned. We define the set of nodes 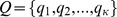 in the graph 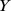 to represent the set of realized hypothetical ancestors for the lineages exemplified in the set of all source tree nodes 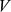. Each node 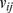 from a source tree 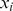 is aligned to at least one node in the graph 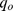. The edges connecting nodes 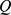 in 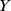 are enumerated in the set 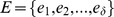.

A least inclusive common ancestor (LICA) is an internal graph node to which internal source tree nodes can be aligned (Figure 1).

### Taxonomic requirements

For multiple trees to be combined, they must all subscribe to a consistent taxonomic naming convention. For simplicity we use labels on the trees as identifiers, though the use of node labels as unique identifiers introduces potential problems regarding lineage identifiability when data from sources using different naming conventions (i.e. taxonomies) are combined. To alleviate these problems, one could use defined identifiers (e.g., global unique identifiers). When using labels to match nodes, all node labels in each input tree are required to be unique. Before source trees are added, the TAG will be loaded with a taxonomy, either with a hierarchy or with no additional information (i.e. where each terminal node is connected to a root node). This provides the graph with the complete set of node labels.

### Overlapping taxon sets

Suppose we want to combine three trees each with the same four taxa: a,b,c, and d (Figure 1A). First, each terminal node in each input source tree is aligned to a graph node based on the node labels. The taxonomy is not shown in Figure 1A to reduce clutter, but is shown as the grey tree in Figure 1B.

After terminal nodes are aligned, internal nodes of each source tree are aligned to nodes identified as LICAs in the TAG. When the taxa are completely overlapping in source trees, a TAG node is a LICA for an internal node in a source tree if the descendants of the graph node (a) include all terminal descendants of the source tree node and (b) does not include any terminal descendants of the source tree excluding those contained within the current source tree node. To state that more technically, we need to define some terms. Let 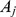 be the set of all subtending nodes arising from an internal source tree node 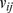. Let 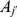 be the set of nodes in the source tree 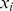 that are not descended from 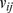. Let 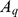 be the set of all subtending nodes arising from a TAG node 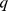. A TAG node in the set of TAG nodes 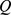 will be a potential LICA if 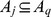 and 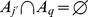. An alternative LICA mapping technique is described below. If no graph node represents a valid LICA for a given source tree node 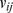, then a new graph node is created and 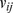 is aligned to it. When mapping the first input tree 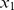 into an empty TAG, a new graph node will be created for every node in 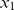, leading to the exact duplication of 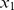 in the graph 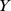. If a LICA is not found a new one is created.

As the set of all input source tree nodes 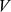 are aligned to LICA graph nodes in 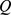, appropriate edges 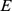 are created. Edges in 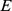 are directed, from children to parents, and individual input branches are mapped directly to one edge (that is, an identical branch from two input trees will be represented by two parallel graph edges). Upon these edges, any amount of branch- or tree-specific metadata may be stored (e.g., branch lengths, branch labels, bibliographic citations, dataset information, inference techniques). Given this structure, the TAG provides lossless storage of all source data. Tree-wise metadata about a source tree may be stored as a property of the source tree root node in the graph. This includes, at a minimum, the set of all terminal node labels that occur in that source tree, but could also include any other information such as provenance, taxonomic scope, authorship, etc. These properties may be queried to return information about (and handles to) source trees stored in the graph, thus providing lossless storage and queryable access to the source trees themselves (within the TAG). With this framework, as demonstrated in Figure 1A, we can align multiple trees with overlapping taxon sets into a graph.

### Partially overlapping taxon sets

In addition to aligning trees with completely overlapping taxon sets, we may align trees with partially overlapping taxon sets (Figure 1B). This example includes a taxonomy (the gray tree) with two internal node names (x and y), the three source trees from Figure 1A, as well as an additional green source tree. For partially overlapping taxon sets, depending on the nature of the overlap and conflict, we can use the procedure described above, or we can align source tree nodes to LICAs in the TAG using slightly modified criteria. This modified criteria includes recording the bipartion, including the information on the taxa subtending the TAG node, as well as the taxa that must be excluded. Therefore the bipartition LICA requirement is as follows. As with above, let 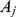 be the set of all subtending nodes arising from an internal source tree node 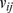 and 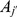 be the set of nodes in the source tree 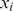 that are not descended from 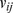. Let 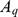 be the set of all subtending nodes arising from a TAG node 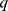 and 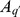 is the set of nodes that are recorded to not be descended from 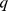. A TAG node is then a potential LICA if 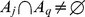, 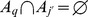, and 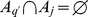. This procedure also requires that all lineages from the source tree be represented in the set of source trees that make up the TAG LICA. The procedure for overlapping taxon sets assumes that any taxa not within a clade are in the other partition. That is not the case for this procedure and so we record what members of the other bipartition have been realized. When there is incomplete overlap in taxa sampled among the source trees the LICAs may partially overlap (e.g., the LICA for a,d in the green tree is the same graph node as the LICA for a,b,c,d in the black, blue and orange trees). It may be the case, with partially overlapping taxon sets, that a node in set of graph nodes 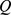 and its parent are both LICAs. In these cases, the LICA is mapped to the shallowest node. It is also possible that multiple LICAs will be found, in which case, each will be mapped. For a specific source tree node this can occur when multiple LICAs contain all of the subtending nodes for the source tree node, but also contain sets of additional nodes that are not present in the source tree.

For partially overlapping taxon sets, the order of trees can influence the alignment of trees into the TAG because of the order of new nodes created. As source trees are aligned into the TAG, graph nodes may be created which represent new LICAs for nodes from previously imported source trees, and in some cases may invalidate LICA mappings for previously added nodes. For example, this can occur when an input tree node 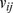 in a source tree 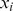 is aligned to a graph node whose subgraph contains terminal nodes not aligned to any nodes in 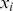. So, 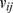 contains a subset of the taxa that are associated with the current LICA(s). A source tree may be added later that necessitates the creation of a new graph node that is aligned to all the descendant terminal nodes of 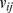, but which is nested shallower than the current LICA of 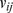. In this case, 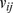 should be re-aligned to the new TAG node. This order-dependence can be overcome, however, by re-aligning the nodes from a source tree. We do this by re-processing source trees once all have been added to determine if there are better LICA mappings, and the final structure is independent of the order of addition. This final graph is what is seen in Figure 1B.

### TAG synthesis techniques

Many operations and queries can be conducted on a graph with trees aligned as shown in Figure 1. One common operation is synthesis of the TAG. By synthesis, we mean the selection of relationships in the graph either by filtering or other procedure to produce a synthetic or composite tree. There are a number of different ways to synthesize the TAG and here we describe a few including (1) preference for specific source trees, (2) preference for more highly supported nodes, and (3) routes with a maximum number of taxa using a branch-and-bound optimization. In Figure 1C, we show the result of one synthetic analysis on the graph where we prefer source trees and specifically (in order) blue, green, and taxonomy source trees.

In each case, synthesis begins by identifying a starting node. For example, this may be a particular clade identified by name or by its set of descendant terminal nodes (i.e. the identification of a LICA). To make a tree from an entire TAG the procedure starts at the root node. From this focal node, it proceeds breadth-first in determining which nodes to include in the synthesis as we traverse the TAG. At each node, the procedure examines the subtending nodes, and determines if any of them conflict. For synthesis, downstream conflict is determined by comparing the LICAs for each child. If the LICAs from nodes subtending the current node overlap, then these descendant subgraphs define incompatible subtrees, and are said to be in conflict (see the sections on measurements of support and conflict for an alternative method of detecting such conflict). In such cases, the procedure must make a decision about which path to prefer. In Figure 1C, we prefer specific source trees, but there are many criteria that can be used to inform these decisions. The resulting synthetic tree in Figure 1C is a composite of the source trees stored in the TAG. Because of the mapping of the source trees to common graph nodes, the synthetic tree includes the internal node names that originate from the taxonomy. Although we present a tree as the result of synthesis, there is no requirement that the synthetic product be a tree. However, a tree will likely be a more common product.

#### Preferring certain source trees

One of the simplest but most useful methods for constructing a synthesized tree is to prefer paths from specific source trees included in the graph. This procedure can generate a tree that is entirely congruent with the most-preferred source tree, but which may contain more lineages. In fact, the synthetic tree will contain terminal lineages from all other source trees in the TAG that are compatible with the preferred set. This procedure requires the identification of a preferred list of source trees, sorted by preference. The trees in the preferred list are consulted in order, and any conflicts among them are resolved in favor of those with higher positions in the list. This procedure could easily be extended to use any kind of source tree metadata, such as pre-calculated node support (e.g. posterior probabilities, bootstrap proportions), presence or absence of branch lengths, or other properties.

#### Preferring better-supported nodes

Properties such as node and edge support can also be used to resolve conflict. In the examples below, node support 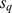 is measured as the proportion of source trees in which the given node is observed. In a TAG constructed from source trees with completely overlapping terminal node sets, the proportion of trees exhibiting a node is the number of outgoing branches (these point to the parent of the specified node). In TAGs containing source trees with incompletely overlapping terminal node sets, node support for a given node 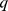 must be corrected to reflect (1) the number of source trees containing any node that may be mapped to 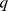, and (2) the potential that the parent of 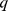 in some source tree could have been aligned to more than one LICA. In this case, 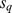 is the number of source trees associated with the set of outgoing edges of 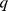, divided by the number of source trees containing any terminal node aligned to any descendant of 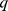 (these are the source trees that could be aligned to 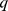).

In a support-based tie-breaking procedure, preference is given to the node with the highest support. In datasets with completely overlapping taxon sets, the nodes chosen by this procedure are frequently the same as those chosen by a traditional consensus tree analysis. However, this is a greedy procedure and does not guarantee that the resulting synthetic tree is the best supported tree. Differences between a majority-rule consensus and a best-support synthesis tree will lie mostly in poorly supported areas of a tree.

#### Preferring complete trees using branch and bound optimization

When constructing a synthetic tree from a TAG containing conflict, one challenge lies in maximizing the number of the terminal nodes that will be present in the final synthetic tree. The worst-case for this problem is presented when no additional information is provided to break conflicts. In a general sense, this is related to a classic set cover problem (Aho et al., 1974), and is likely to be NP-complete. The solution can be greatly simplified by specifying other properties (such as those mentioned in the previous two sections) with which to break conflicts. In lieu of such specified properties, however, we present a branch-and-bound approach to attempt to maximize the number of terminal nodes in the synthesized tree. There are two implementations of this algorithm: in the first, the bound is based on minimizing cost; in the second, it is based on maximizing scores. The algorithms are presented in the supplemental materials.

### Measuring support and conflict within a TAG

In simple examples as in Figure 1, conflict and support is easy to observe. For more complex TAGs, such as the angiosperm TAG described below, we can calculate node- and edge-based statistics on the graph 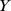 to describe support and conflict throughout. Node support is calculated as the number of source trees aligned to the focal graph node, scaled by the number of source trees that could be aligned to that node (i.e. contain overlapping taxon sets). Edge support, 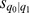 for the edge between the child node 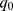 and its parent 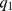, is calculated in a similar fashion to node support: it is the number of times that edge is observed (i.e. the number of exactly parallel graph edges between the same parent and child nodes) scaled by the number of source trees in which that edge may be observed.

Complementary to edge-based metrics of support, node-degree (the number of nodes adjacent to a focal node) reflects node-based conflict and uncertainty. As a TAG is a directed graph, we partition node-degree into 1) child- and 2) parent-node relationships. Simple child- or parent-degree counts are not directly informative, as node relationships can be supported to different extents (i.e. by the number of source trees exhibiting the relationships). Because all node relationships from all input source trees are preserved in the graph (even identical relationships), we can instead calculate the *effective* number (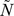) of directed node relationships. Consider a focal graph node 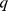 with 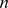 parent nodes, but 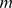 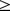 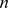 parent-node relationships (that is, 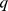 is present in 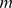 source trees, all of which may potentially differ in taxon overlap). Each parent node 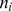 is supported by some proportion 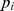 of the 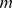 trees. The effective number of parent nodes for 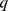 is given by:
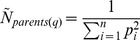
(1)


The effective number of child nodes 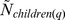 is calculated similarly, dealing with the number of child nodes and child-node relationships. This metric corresponds to the inverse Simpson diversity index (Simpson, 1949), and is larger when directed node relationships are more evenly supported. For example, a graph node with two parent nodes each supported by 500 trees will have 2 effective parents, whereas another graph node with one parent supported by 999 trees and another parent supported by 1 tree will have 1.002002 effective parents. Note that terminal graph nodes possess no children, and the root graph node has no parent. In general, 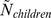 reflects phylogenetic uncertainty (or lack of information), while 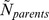 reflects immediate topological conflict (e.g. when a node has 2 or more parent nodes; Figure 1A). In a fully bifurcating TAG, each internal node will have 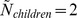 and 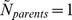. Note that in the tree synthesis procedures above, an overlap of LICA descendants indicates that one or more topological conflicts (that is, 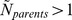) resides somewhere within the subtending graph.

While the effective number of parents indicates the degree of immediate topological conflict, it ignores the frequency at which a given node 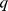 occurs across a set of source trees. We therefore also define a measure of destabilization, which is the number of effective parents for node 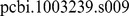 scaled by its support:
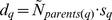
(2)


Destabilization measures the contribution of a given LICA to the conflict in the neighboring nodes. Graph nodes with high values of 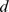 are frequently observed in source trees but rarely in the same topological position. These nodes contribute heavily to the collapse of clades in traditional consensus methods.

For each internal node 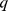 in the graph we also calculate the average 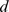 for all 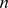 descendant nodes:
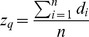
(3)


A 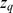 value greater than 1 indicates that the subgraph of 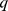 contains conflict. This statistic has the useful property of being directly comparable among TAGs constructed from different data sets.

It is also useful to quantify how average downstream conflict 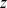 changes with the inclusion of a graph node 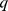 and its descendant subgraph. We therefore compute a metric of resolution, which quantifies the difference in average downstream conflict between a node 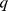 and its 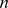 immediate parent node(s):
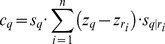
(4)where 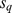 is the node support for node 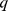, 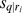 is the edge support connecting node 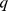 to its parent node 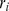, and 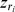 is the average downstream conflict experienced by parent node 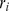. High values of 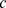 indicate clades that are frequently observed but whose inclusion in the graph contributes heavily to overall conflict within their parent clade.

### Implementation

These methods have been implemented in the software treemachine, which makes use of the NoSQL graph database Neo4j (http://www.neo4j.org/). Treemachine is an open source application developed for the Open Tree of Life project (http://opentreeoflife.org/). The source code and executable used for the examples presented here are available from the treemachine repository on GitHub https://github.com/OpenTreeOfLife/treemachine.

## Results/Discussion

### Empirical example datasets

We demonstrate the methods described above using two empirical datasets. First, we use bootstrap trees for 640 species representing the majority of known seed plant lineages from the Angiosperm Tree of Life analyses [23]. These bootstrap samples are used to demonstrate the utility of TAGs for exploring conflict within a dataset of completely overlapping taxa. Second, we explore a bird dataset analyzed in Jetz et al. [19] that includes source trees for individual clades of birds, a backbone phylogeny, and taxonomic information. We use this to show how TAGs may be employed for tree-grafting procedures that can combine less-inclusive trees to create more-inclusive ones. Data used in this study has been deposited in Dryad (http://datadryad.org/).

### Mapping uncertainty in seed plant phylogeny

The methods discussed here can be useful for exploring conflict and congruence among source trees with completely overlapping terminal nodes (e.g. a bootstrap or posterior distribution of trees). We explore this on the Angiosperm Tree of Life dataset [23]. This dataset is one of the most comprehensive phylogenetic datasets available for flowering plants, and represents confidence, or lack thereof, in flowering plant relationships. Although relationships in many clades have been well described, relationships within some have proven difficult to resolve. The conflict introduced by these unresolved clades provides an ideal case for the exploration of TAGs as a datatype to investigate uncertainty.

**Figure 2:**
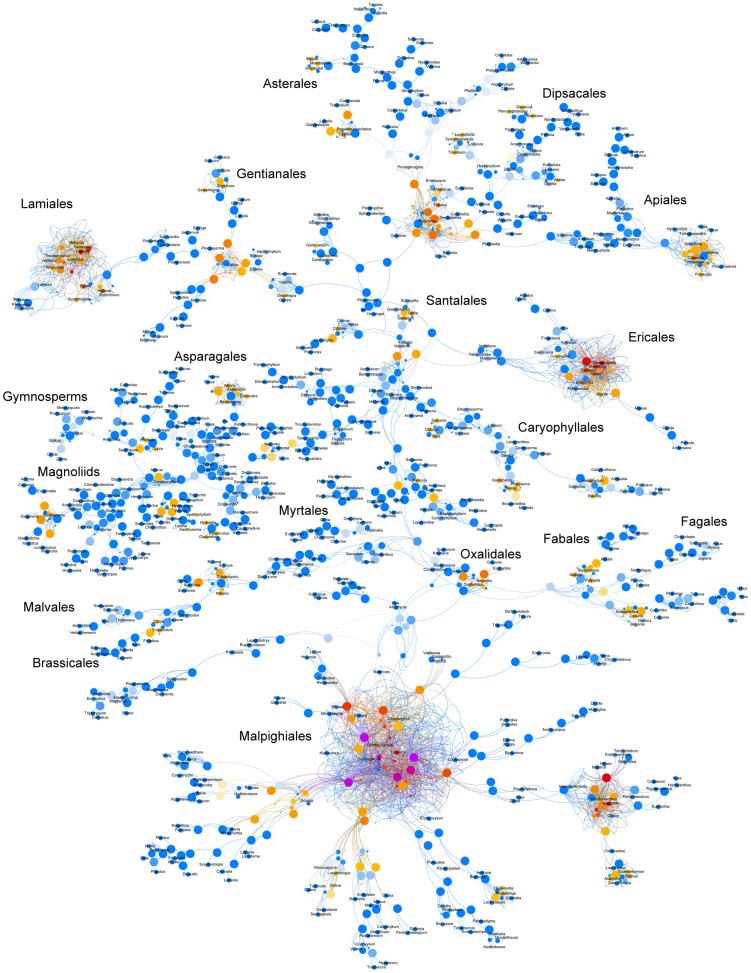
Graph of Angiosperm Tree of Life with effective parents. A graph showing the mapping of the Angiosperm Tree of Life project bootstrap dataset of 100 trees that includes 640 taxa with chloroplast, mitochondrial, and ribosomal data [23]. Larger node size indicates higher node support. Node color indicates the number of effective parents (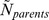) of each node, with blue nodes having relatively small values of 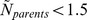, orange nodes with values between 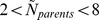 and red to pink nodes with values of 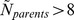.

**Figure 3:**
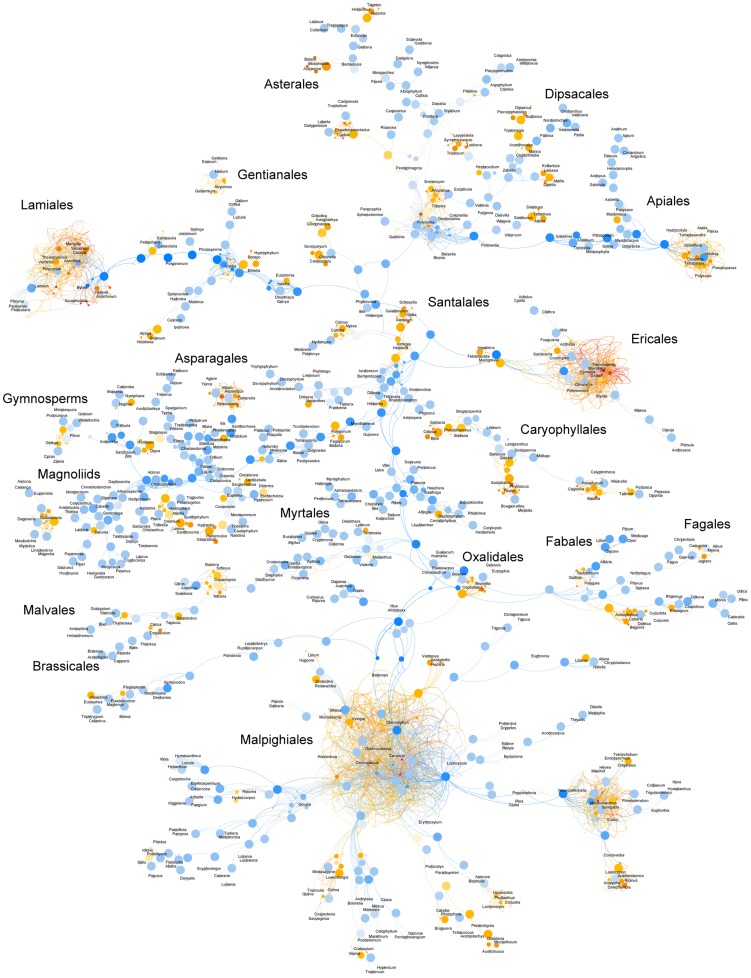
Graph of Angiosperm Tree of Life with average effective parents. A graph showing the mapping of the Angiosperm Tree of Life project bootstrap dataset of 100 trees that includes 640 taxa with chloroplast, mitochondrial, and ribosomal data [23]. Larger node size indicates higher node support. Node color indicates the average subgraph destabilization (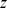) of each node, with blue nodes having relatively small values of 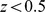, orange nodes with values between 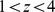 and red to pink nodes with values of 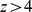.

**Figure 4:**
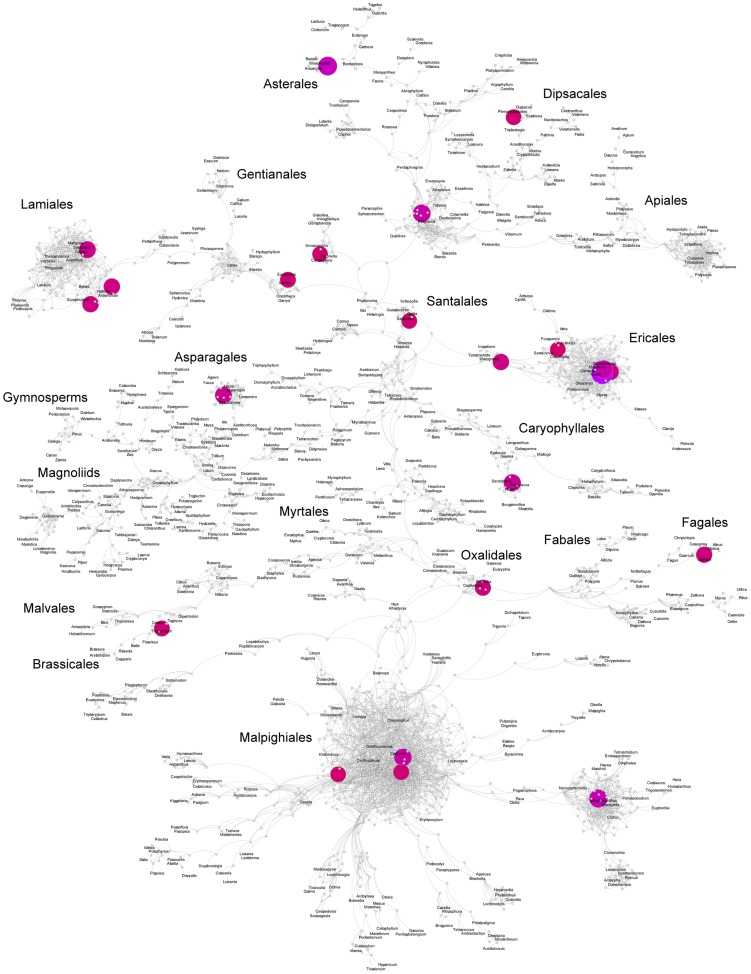
Graph of Angiosperm Tree of Life with nodes having the highest amount of conflict highlighted. A graph showing the mapping of the Angiosperm Tree of Life project bootstrap dataset of 100 trees that includes 640 taxa with chloroplast, mitochondrial, and ribosomal data [23]. Large purple nodes are those corresponding to nodes in Table 2.

The TAG was constructed by loading one hundred bootstrap trees constructed with the full dataset of all 17 gene regions and 640 taxa included in the original study. No additional structural information (e.g. a taxonomy tree) was given. Figures 2–4 depict this graph. Much of the structure of the graph is resolved (denoted by the large proportion of blue nodes in Figure 2), in accordance with the consensus tree presented in the original publication. Though some areas have significantly more complex structure (denoted by the large complex reticulate structures).

To characterize conflict within this TAG we use several statistics described above including node support (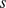) and the effective number of parent nodes (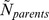). Highly supported nodes with a low number of effective parents (i.e. high 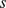, low 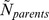), represent nodes that are frequently recovered and confidently placed in the source trees (large blue nodes in Figure 2). Highly supported nodes with high 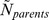 (large, orange to pink) are nodes that are frequently resolved in source trees but their placement varies among bootstrap replicates. We calculated destabilization statistics (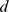) for this TAG as well. Nodes with high support and low confidence have high 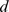 and represent major sources of conflict, whereas nodes with low 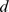 contribute little to overall conflict. Table 1 presents the nodes with the highest 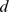 values in the dataset. Note that a high proportion of destabilizing nodes are from the order Malpighiales.

**Table 1:** Twenty-five destabilizing plant clades.

rank	order	taxon	*s*	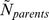	*d*
1	Malpighiales	Lophopyxidaceae + Putranjivaceae	1.00	18.38	18.38
2	Malpighiales	Pandaceae	1.00	18.25	18.25
3	Malpighiales	Salicaceae + Lacistemataceae	0.99	17.22	17.05
4	Malpighiales	Picodendraceae + Phyllanthaceae	0.99	15.43	15.28
5	Malpighiales	Malpighiaceae + Elatinaceae	1.00	15.02	15.02
6	Malpighiales	clade 1	1.00	9.75	9.75
7	Ericales	clade 2	0.98	9.42	9.23
8	Malpighiales	Linaceae	1.00	7.52	7.52
9	Malpighiales	clade 3	0.48	15.16	7.28
10	Malpighiales	clade 4	1.00	6.95	6.95
11	Malpighiales	Euphorbiaceae + Ochnaceae	1.00	6.79	6.79
12	Bruniales	Columelliaceae	1.00	5.26	5.26
13	Bruniales	Bruniaceae	1.00	5.25	5.25
14	Oxalidales	Cunoniaceae	1.00	5.21	5.21
15	Lamiales	Martynia + Sesamum	0.61	8.40	5.12
16	Escalloniales	Escalloniaceae	0.91	5.47	4.98
17	Solanales	Solanales	1.00	4.78	4.78
18	Malpighiales	Ochnaceae	1.00	4.74	4.74
19	Asterales	clade 5	1.00	4.69	4.69
20	Gentianales	Gentianales	1.00	4.66	4.66
21	Lamiales	Lamiales	1.00	4.44	4.44
22	Malpighiales	Manihot + Hevea	1.00	4.34	4.34
23	Malpighiales	clade 6	0.96	4.42	4.24
24	Oxalidales	Elaeocarpaceae	0.97	4.20	4.08
25	Apiales	Apiales	1.00	4.03	4.03

Top twenty-five nodes with the highest 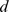 scores, representing destabilizing clades. These clades are relatively well-supported but their placement in the tree is uncertain. Clades that are too large for the table are listed here. Clade 1: Tetrorchidium + Omphalea + Endospermum. Clade 2: Ericaceae + Clethraceae+ Cyrillaceae + Sarraceniaceae + Actinidiaceae. Clade 3: Rhizophoraceae + Erythroxylaceae + Ctenolophonaceae. Clade 4: Chrysobalanaceae + Euphroniaceae + Dichapetalaceae + Trigoniaceae + Balanopaceae. Clade 5:Asteraceae + Calyceraceae + Goodeniaceae + Menyanthaceae + Stylidaceae + Alseuosmiaceae + Argophyllaceae + Phellinaceae + Campanulaceae + Rousseaceae. Clade 6: Homalanthus + Euphorbia + Pimelodendron + Hura.

We also summarize the information regarding destabilization for descendent subgraphs. Figure 3 presents the same information as in Figure 2, except that color corresponds to the average destabilization for all descendant nodes (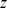). 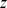 is large (orange to pink color) when the descendant subgraph has a large amount of conflict, and small (blue) when the descendant subgraph has little conflict. This statistic is helpful for identifying when a clade originating from a particular node contains significant conflict.

These graph visualizations facilitate a rapid assessment of patterns of phylogenetic conflict, and can reveal details that are not as easily discerned with traditional consensus methods. Regions of the tree that are characterized by high conflict are indicated in the graph as highly reticulated subgraphs or ‘hairballs’ (Figures 2 and 3). The three largest of these problem areas in this dataset correspond to the clades Malpighiales, Lamiales, and Ericales. Other, smaller areas of conflict include regions in the Apiales, Asparagales, Asterales, Dipsacales, Escalloniales, Caryophyllales, Fabales, Ranunculales, Santalales, and Solanales. In Figure 2, these areas are characterized by the presence of bright orange to pink (high 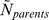) nodes, whose placement is uncertain. When nodes of uncertain placement are observed in a large proportion of trees (i.e. highly supported nodes; large orange to pink nodes in Figure 4), the differences in their phylogenetic position cause a proliferation of relationships, leading to the reticulated regions. In typical consensus trees, these areas are indicated by the presence of polytomies and overall low support values. However, typical consensus trees display only edges occurring in some minimal proportion of input trees (e.g. 50% in majority-rule consensus trees). In such summary trees, equally-supported but conflicting edges in the source trees, as well as bipartitions slightly less frequent than the minimum threshold (e.g. node 9 from Table 1), are never displayed.

To assess overall conflict within clades, we calculated 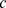, a measure of phylogenetic conflict within the descendant subgraph, scaled by its support, 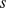. This differs from the 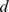 statistic in incorporating the conflict within the subgraph. High values of this statistic indicate nodes that contribute heavily to the overall level of conflict within their parent clade. This statistic is useful for identifying putative clades with the highest overall levels of conflict in the tree. Table 2 identifies the 25 nodes with the highest values for 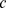. Figure 4 identifies the precise location of these nodes within the graph.

**Table 2:** Twenty-five plant clades with low resolution.

rank	order	taxon	*s*	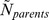	*z*	*c*
1	Ericales	Sapotaceae + Pentaphylacaceae	0.18	8.53	10.19	1.32
2	Asterales	Calyceraceae	1.00	1.13	2.08	0.97
3	Ericales	Theaceae + Pentaphylacaceae	0.13	3.45	9.59	0.92
4	Malpighiales	Suregada + Moultonianthus	0.18	4.91	7.13	0.90
5	Ericales	Theaceae + Ebenaceae	0.27	6.45	5.76	0.86
6	Escalloniales	Polyosma + Tribeles	0.35	1.00	3.51	0.82
7	Malpighiales	Caryocar + Octhocosmus	0.16	8.53	6.84	0.78
8	Caryophyllales	Phytolaccaceae + Sarcobataceae	0.36	1.48	3.74	0.74
9	Asparagales	Asparagus + Beaucarnea	0.50	2.13	3.11	0.69
10	Lamiales	Martynia + Sesamum	0.61	8.40	2.58	0.68
11	Ericales	Diapensiaceae + Styracaceae	0.41	3.68	3.93	0.65
12	Lamiales	clade 1	0.28	1.24	3.00	0.62
13	Ericales	clade 2	1.00	1.11	1.23	0.62
14	Lamiales	Scrophulariaceae + Stilbaceae	0.19	1.36	4.26	0.62
15	Fagales	Juglandaceae + Myricaceae	0.58	1.07	2.04	0.59
16	Oxlidales	Brunelliaceae + Cephalotaceae	0.44	2.35	3.05	0.57
17	Malpighiales	Caryocaraceae + Putranjivaceae	0.16	9.14	5.14	0.55
18	(asterids)	Garryales + Icacinaceae	0.69	1.66	1.46	0.53
19	Brassicales	Caricaceae + Tropaeolaceae	0.42	1.00	2.40	0.53
20	Dipsacales	Dipsacus + Pterocephalodes	0.54	1.00	2.26	0.52
21	Santalales	Santalaceae + Opiliaceae	0.28	1.00	3.42	0.52
22	Malpighiales	Irvingiaceae + Ixonanthaceae	0.38	1.69	2.51	0.51
23	Aquifoliales	Cardiopteridaceae	0.97	1.00	1.54	0.50
24	Ericales	Sarraceniaceae + Actinidiaceae	0.73	1.00	1.68	0.49
25	Malpighiales	Acalypha + Spathiostemon	0.54	1.50	2.63	0.47

Top twenty-five nodes with the highest 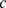 scores, indicating clades with relatively low phylogenetic resolution compared to surrounding parts of the tree. Clades that are too large for the table are listed here. Clade 1: Plantaginaceae + Scrophulariaceae + Stilbaceae. Clade 2: Tetrameristaceae + Balsaminaceae + Marcgraviaceae.

**Figure 5:**
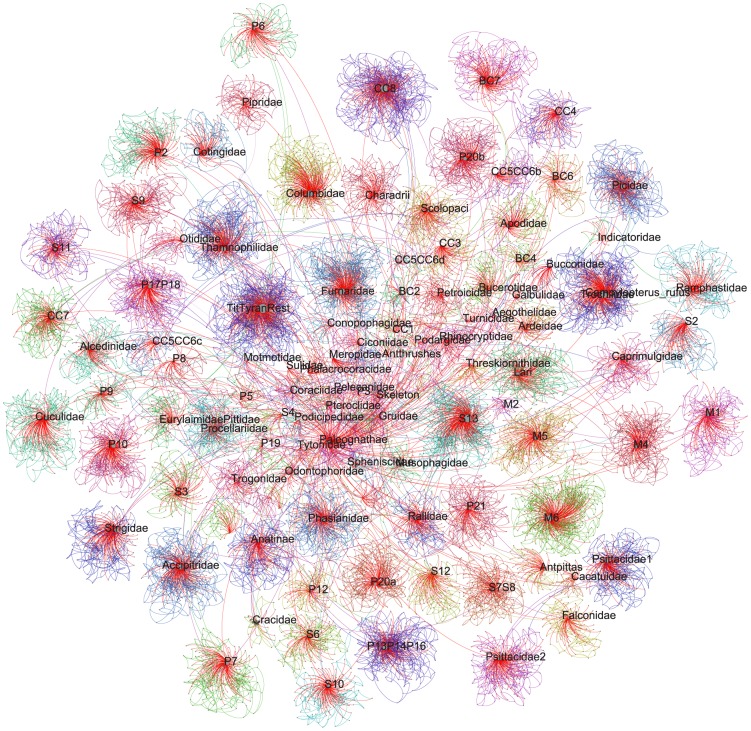
Avian tree alignment graph. A graph showing the relationship of birds using input source trees from Jetz et al. [19]. Each edge color is a different source tree with the red being taxonomy. The names at the internal nodes are scaled by the number of edges and refer to those in the taxonomy from the original reference.

Specific patterns within areas of conflict differ among major clades. In Malpighiales, for instance, the relatively low frequency of nodes in Table 2 compared to Table 1, as well as the high proportion of blue nodes in this clade in Figure 5, indicates that despite the relatively high level of conflict regarding the placement of several Malpighialean nodes (Table 1), relationships within this clade are better known than those in other areas of the tree. Only a few large, pink nodes in Malpighiales in Figure 2 appear to be driving the conflict within the clade as a whole. This contrasts with the patterns in Lamiales and Ericales, where little agreement exists among source trees regarding the placement of most nodes, as indicated by the high proportion of orange to pink nodes in Figure 3. Many of these nodes have support values of less than 0.5 (Table 2), indicating that they will never appear in majority-rule consensus trees. Indeed, overall support values throughout these clades are low, suggesting little to no clear phylogenetic signal within these groups. Numerous other areas with relatively high levels of phylogenetic conflict can be identified with the TAG (Table 2; smaller reticulated subgraphs with orange to pink nodes in Figure 3) and represent uncertain regions of the dataset and that may benefit from additional sampling.

TAGs provide a means for analysis and visualization of uncertainty and alignment of trees. As demonstrated, in addition to edge-based indices of support (analogous to consensus tree bipartition support), TAG-specific metrics (e.g. 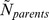) can more extensively quantify phylogenetic conflict. The information presented in Figures 2 and 3, and Tables 1 and 2 reveals a detailed picture of the patterns of conflict within the seed plant phylogeny. The high proportion of large blue nodes in both Figures 2 and 3 indicates that the major structure of the graph is well resolved, but several areas of high conflict are easily noted and can be explored in great detail (Figures 2–4 and Tables 1 and 2). The clades identified in Table 2 based on the 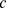 statistic represent the areas of angiosperm phylogeny in which we are least confident, and as such are those regions with the highest potential for improvement. Their identification should inform future sampling efforts to target these groups.

The information summarized by the statistics calculated on the TAG is challenging to obtain with traditional consensus methods but is easily browsed and queried within a TAG. Although, the results from the TAG analyses agree with the interpretation of these data in Soltis et al. [23], the TAG and the graph-based statistics allow for a more flexible, detailed, and targeted exploration of these patterns. The use of TAGs provides a complementary approach to traditional consensus methods, facilitating the analysis and visualization of this information in powerful and detailed new ways. In particular, as demonstrated, measures such as destabilization and other subgraph summaries can assist in identifying nodes that need more attention or data collected. Although not explored here, researchers could load tree sets from different analyses, and even partially overlapping taxon sets, to explore the same statistics and perform similar analyses.

### Synthesizing the avian TAG

The methods discussed here are not only useful for examining uncertainty in datasets with fully overlapping taxon sets, but also can be used for exploring trees with partially overlapping taxon sets. In particular, the graph procedures described perform well on datasets with very hierarchical strucuture. One of the most ambitious manual tree grafting exercises performed to date involved the recent complete phylogeny of birds [19]. Combining a set of partially-overlapping clade-specific trees with a comprehensive taxonomy, Jetz et al. [19] were able to construct a phylogeny of all extant bird species, despite many taxa lacking genetic data. This type of analysis can be very useful when conducting evolutionary analyses at large taxonomic scales, as sampling in molecular phylogenies is often incomplete. The type of grafting used by Jetz et al. [19] not only allows for the inclusion of taxa not sampled in molecular phylogenies, but also takes advantage of the phylogenies constructed at deeper phylogenetic levels to join phylogenies constructed at shallower levels. One challenge of manual tree grafting exercises is the process of updating based on new or different source trees as well as repeatability of the synthesis of the generated trees. Another challenge is, when there is conflict, communicating the specific algorithmic decisions for repeatability or updating with new data [24]. The Jetz et al. [19] dataset serves to demonstrate the strength of the methods presented here in automating the alignment and synthesis of trees with different taxonomic levels, and with different levels of completeness.

To construct the TAG we first used the taxonomy defined by the original publication that consists of species names and their placement within broader clades (genera, families, orders, and higher). These species are meant to represent all known extant bird diversity. For the 6800 taxa that possess some genetic data, we constructed maximum clade credibility (MCC) trees for each of the 129 clade-specific datasets defined by Jetz et al. [19] (available at http://birdtree.org/). The entire posterior distribution, or samples thereof, could be added instead of a MCC tree; however, to allow for a simpler graph presentation in this proof-of-concept exercise, we restricted consideration to MCC trees. As in the original study, in addition to the clade-specific source trees, we also employ the backbone phylogeny based on the Hackett et al. [25] study. The backbone phylogeny and each of these 129 clade-specific MCC trees were added to the graph of the taxonomy (Figure 5). We then updated the mapping of the source trees based on the addition of potentially new least inclusive common ancestors.

**Figure 6:**
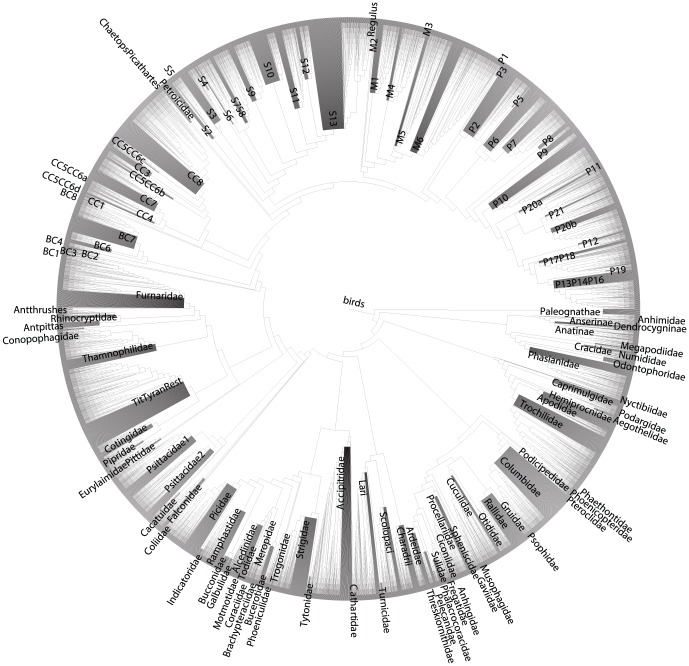
Synthetic avian phylogeny. The synthetic tree produced from processing the graph of bird source trees and taxonomy from Jetz et al. [19] presented in Figure 3. Names at the internal nodes represent the larger taxonomic groups as recognized by the original publication. The tree is the product of each of the source trees and the taxonomy. The large blocks represent species that are unresolved but present in the taxonomy.

The final TAG (Figure 5) represents the taxonomy, backbone tree, and all 129 clade-specific MCC trees. To demonstrate the ability to construct a grafted tree from this final TAG, we constructed a synthetic tree (Figure 6). In order to construct a synthetic tree on the TAG, the criteria for resolution of conflict needs to be established. The criteria used for this example favored (in order) the Hackett et al. [25] backbone, each of the trees based on genetic trees where available, and taxonomy. The synthetic tree (Figure 6) contains some taxa that are only present in the taxonomy and are represented as large polytomies. Unlike the original publication, we do not resolve these branches. This could be done but is not specifically part of the graph procedure, and so we do not conduct that additional analysis here. The alignment and synthesis analyses takes a relatively short amount of time despite the number of trees and the size of the dataset (<2 minutes on a quad-core Xeon 2.67 GHz desktop). This relatively simple analysis demonstrates the ability of the methods discussed here to be applied to the problem of constructing large phylogenies based on source trees with only partially overlapping taxon sets. The automated nature of this analysis makes for easy updating with new source tree information, accommodation of uncertainty, and a regular and repeatable way to construct additional synthetic trees based on different criteria.

In addition to helping visualize and analyze conflict among sets of trees, the avian example demonstrates the utility of TAGs for automated synthesis of trees. The synthesis performed can be communicated easily, performed algorithmically, and repeated. Unlike some supertree methods, it is also easy to trace each branch that is recovered in the final synthesis to the original source tree(s) that supports it. Furthermore, in both synthesis and conflict analysis, the graph can continue to be updated with additional source trees (e.g., from additional bootstrap, Bayesian analyses, or other sources).

### Comparison to existing methods

There is a rich literature of methods and approaches for accommodating reticulation events in phylogenetics that fall under the title phylogenetic networks [11]. A large number of these approaches are implemented in the program SplitsTrees [4] and Dendroscope [26]. Many of these procedures are intended to use the sequence data to infer structures other than trees. This is notably different than the methods presented above where we aim to align trees into a common structure that may require the creation of nodes that have conflict. The methods presented here also differ from those generally considered to be rooted phylogenetic networks [11]. Those methods are intended to infer network structures instead of aligning trees into a general graph. The graph methods presented here address a different problem than existing phylogenetic network methods.

More recently, Berry et al. [18] introduced a graph solution to combining trees of partially overlapping taxa at different taxonomic levels. Our approach differs in key aspects. Their MLS (MultiLevelSupertree) is restricted to returning a single supertree compatible with all source trees. It is not presently possible to ascertain the degree of topological conflict involved in the production of a MLS supertree. In addition, graphs in the MLS method exist only during runtime, and so cannot be distributed or augmented without rerunning the amalgamation steps as presented in their manuscript. MLS graphs also differ in that they contain both descendant and non-descendant (sibling) edges (the latter not represented in any of the source trees), and so are not as amenable to interpretation, querying, or visualization. Finally, unlike the MLS method, our approach retains all source tree information (e.g. branch lengths, node support), including an arbitrary number of tree annotations (e.g. genome, tree reconstruction method, etc.). We believe such metadata will feature prominently in many customized user queries of phylogenetic tree graphs.

The graph framework presented here provides an accurate representation of the input source trees, methods for identification of congruence and conflict among source trees, methods for synthesis of source trees, and efficient storage that can be queried and scale adequately with the ever increasing number and size of present-day phylogenetic trees. The set of algorithms described here can be used to map any set of evolutionary trees or hierarchies (source trees) into a common graph, while retaining all of the phylogenetic information present in the source trees. The methods discussed here are implemented in a graph database. By utilizing a database for the storage of this information, queries, visualizations, and synthesis using a large number of different methods can take advantage of shortened runtime and frequent updates. Importantly, each analysis does not require reanalysis of the source trees.

### Other applications

The methods presented here are agnostic to data source. The underlying model assumes only that the input trees define ancestor-descendant relationships. Neither do the source trees need be derived from the same dataset, nor do their tip sets need to extensively overlap. Terminal nodes in source trees represent lineages (e.g. species, populations, alleles, etc.). However, the terminal nodes need not be extant, nor reside at the same hierarchical level-terminal nodes from a given input tree may correspond to internal nodes in another. In the implementation we present, the set of all labels must be pre-identified, and terminal nodes from input trees with labels not in the pre-identified set are not added to the graph. This constraint, however, is not inherent in the procedures themselves. Individual applications of TAGs could vary these requirements for specific purposes. As a result, the methods presented here could potentially be applied and adopted for a wide range of applications in evolutionary biology and phylogenetics. For example, as demonstrated with the avian dataset, taxonomic hierarchies can be combined with phylogenetic datasets. Phylogenetic trees based on morphological data and/or phylogenetic trees of extinct taxa can be easily combined with phylogenies built using extant and molecular data. Other hierarchies could be incorporated into these graphs for analysis and synthesis.

### Conclusion

TAGs provide a means to examine conflict, conduct phylogenetic synthesis, develop complex queries, and allow for detailed visualization. The methods presented here can scale to millions of nodes and can be extended in several ways. However, these methods are not meant to be exhaustive. There are potentially many ways of parameterizing a graph and aligning trees into a graph. Nevertheless, by aligning sets of trees into a graph that can be queried and that retains all the source tree information, we can conduct powerful analyzes on enormous datasets.
